# Assessing Stress in Pregnancy and Postpartum: Comparing Measures

**DOI:** 10.1007/s10995-020-02978-4

**Published:** 2020-07-20

**Authors:** Irena Štěpáníková, Elizabeth Baker, Gabriela Oates, Julie Bienertova-Vasku, Jana Klánová

**Affiliations:** 1 Research Centre for Toxic Compounds in the Environment (RECETOX), Masaryk University, Brno, Czech Republic; 2 Sociology Department, University of Alabama at Birmingham, 1401 University Blvd. HHB, Birmingham, AL USA; 3 Department of Pediatrics, Division of Pulmonary and Sleep Medicine, University of Alabama at Birmingham, Birmingham, AL USA; 4 Department of Pathological Physiology, Faculty of Medicine, Masaryk University, Brno, Czech Republic; 5 Denisa Ludvikova Vizentova, Masarykova univerzita Kotlářská 2, Brno, 61137, Czech Republic

**Keywords:** Pregnancy, Postpartum, Maternal stress, Life events, Child psychological adjustment

## Abstract

**Introduction:**

Measuring early-life psychosocial stress is complicated by methodological challenges. This paper compares three survey instruments for the assessment of life in pregnancy/postpartum and investigates the effects of the timing of early-life stress for emotional/behavioral difficulties (EBD) of offspring during mid/late childhood and adolescence.

**Methods:**

Observational data were obtained from the European Longitudinal Cohort Study of Pregnancy and Childhood (ELSPAC-CZ), which included 4811 pregnancies in two Czech metropolitan areas. We used data collected between 1991 and 2010 at 20 weeks of pregnancy (T1), after delivery (T2), at 6 months postpartum (T3), and at child’s age of 7 years (T4), 11 years (T5), 15 years (T6), and 18 years (T7). Life stress was assessed with (1) the Edinburgh Postnatal Depression Scale (EPDS), (2) a stressful life events (SLE) count based on 42-item inventory, and (3) the SLE measure weighted by perceived stressfulness (PS). Each stress measure was administered at T1, T2, and T3. Child’s EBD were assessed with the Strengths and Difficulties Questionnaire at T4, T5, T6, and T7.

**Results:**

Each stress measure independently predicted long-term EBD. The best data fit was obtained in a model combining EPDS and SLE. Effect sizes for SLEs decreased between the first half of pregnancy and postpartum, while the effect of EPDS increased.

**Discussion:**

SLE-based methods capture an aspect of perinatal stress not adequately assessed by EPDS. Combination of psychological distress measures and SLE-based measures is optimal in predicting EBD of the child. Stress measures based on SLE are suitable for early pregnancy, while self-reports of depressive symptoms may perform better in postpartum.

## Significance

*What is known on this subject*? Perinatal stress contributes to psychological difficulties in later childhood. Instruments specifically designed to measure psychosocial stress during pregnancy/postpartum are scarce, and most studies rely on instruments designed to assess psychological distress, e.g., depressive symptoms.

*What does this study add*? This prospective population-based study investigates the predictive value of three measures of maternal life stress applied at three different times during pregnancy and postpartum. Perinatal life stress is a multi-faceted phenomenon best captured by a combination of instruments. Stress measures based on life events are particularly useful during pregnancy, while measures of depressive symptomatology perform better in postpartum.

## Introduction

Although it is widely acknowledged that stress in utero and during early childhood contributes to psychological problems in the later life of the child, measuring early-life stress is complicated by methodological challenges (Cheung 2002; Gunnar 2015; MacKinnon et al. 2018; Rice et al. 2007; Sourander 2016; Weinstock 2008). This paper examines survey-based methods of life-stress assessment in an observational study of pregnant and postpartum women. Survey-based research is cost-effective and suitable for large population studies. Yet, survey instruments specifically designed to measure stress during pregnancy/postpartum are scarce, and most investigations rely on instruments designed to assess psychological distress, e.g., depressive symptoms (DS) (Gonzalez-Ochoa et al. 2018).

Interpreting psychological distress as a measure of overall life stress has limitations. Stress is conceptually distinct from depression, anxiety, and other forms of psychological distress. In 1930s, Selye defined stress as an organism’s non-specific response to any demand for change. He distinguished stress from stressors, defined as noxious stimuli causing stress (Selye 1998). Despite criticism (Brosschot et al. 2018), Selye’s definition facilitated the conceptualization of stress as a general adaptation. Selye focused on the physiology of stress response through the hypothalamic–pituitary–adrenal (HPA) axis and the sympathetic-adrenal-medullary system. Although his definition did not exclude psychological responses, later scholarship argued that these responses must be incorporated explicitly. Lazarus and Folkman (1984), for instance, defined stress as any situation where perceived demands exceed one’s capacity to adapt, emphasizing the interpretive aspects of stress. In psychologically-oriented approaches, human emotions, such as fear, anger, and sadness, are considered integral parts of stress rather than stress correlates (Lazarus and Folkman 1984).

Psychological approaches remain popular despite the circularity problem inherent in incorporating psychological distress into the conceptualization of stress (Lazarus and Folkman 1986). When the distinction between stress and psychological distress is obscured, the understanding of their relationships is necessarily hindered. This limitation is serious as considerable heterogeneity exists in psychological responses to stressors by genomic/epigenomic makeup and environmental circumstances (Bonanno and Mancini 2012). Some individuals maintain good mental health even under severe, long-term stress. Conversely, psychopathology can develop when measurable stress is absent (e.g., endogenic depression).

Conflation of stress with psychological distress is common in life-stress research. Measures of general psychological distress or stress-related reactivity are often used to assess stress exposure, and “constructs that are not life stress (e.g., sleep problems, depression)” are interpreted as indicators of stress (Slavich 2019, p. 2). Such practices are particularly troublesome when a focus of the study is on pregnant and postpartum women. During pregnancy/postpartum, massive neuro-endocrine-immune changes take place, influencing mental states. Pregnancy in itself challenges adaptation as evidenced by increased hypothalamic–pituitary–adrenal activation and physiological hypercortisolism (Abdelmannan and Aron 2011). Point prevalence of major/minor depression during pregnancy and the first year postpartum nears 13% (Gavin et al. 2005). These considerations suggest that relationships between exposure to stressors and psychological distress may vary between pregnant/postpartum women vs. general population, challenging the use of indicators of psychological distress as proxies for stress during pregnancy/postpartum.

This study investigates the use of surveys to assess life stress during pregnancy/postpartum. The objective is to compare three instruments specifically designed for pregnancy/postpartum, clarifying their conceptual basis and empirical utility. For consistency with prior research, we include the Edinburgh Postnatal Depression Scale (EPDS, Cox et al. 1987), designed to assess postpartum depressive symptoms (DS) but often interpreted as an indicator of stress. Its use is consistent with the view that emotional dysregulation is an integral aspect of stress; a variant justification assumes that emotional dysregulation and stress are conceptually different but empirically correlated; therefore, observed emotional problems accurately represent unobserved levels of stress. Since we consider these assumptions problematic, we compare EPDS to two instruments that assess stress directly using a stressful life event (SLE) inventory. Following Holmes and Rahe (1967), we conceptualize life events as stressors assuming that they demand social adjustment, and exposure to life events as stress. The first instrument, rooted in approaches that focus on exposure to stressful stimuli (Butler 1993), operationalizes stress as a SLE count during a specified period. The assumption is that the degree of stress corresponds to the degree of exposure to stressors, measured as the SLE. The second SLE-based instrument adds a subjective dimension by weighing life events by their perceived stressfulness (PS). This instrument assumes that stress levels correspond to individual interpretations of stressors currently present in one’s life.

To understand the empirical utility of each stress instrument, we investigate their relationship with psychological adjustment in children aged 7–18 years. Based on evidence of adverse effects of early stress exposure on the developing brain and nervous system (Buss et al. 2010; Mareckova et al. 2018; Qiu et al. 2015), we expect that pregnancy/postpartum stress will predict emotional/behavioral problems in childhood. We also address the timing of stress, which often is neglected or bracketed into overly broad categories (Slavich 2019). The timing of stress is important during the perinatal period, when organs and systems develop rapidly and are vulnerable to adverse environmental influences. To provide a nuanced understanding, we use repeated measurement of stress at three perinatal time points.

## Methods

### Data Statement

Data may be requested at www.elspac.cz.

#### Sample

Data were obtained from the Czech part of the European Longitudinal Cohort Study of Pregnancy and Childhood (ELSPAC-CZ). ELSPAC-CZ was approved by the Scientific Committee of Masaryk University for compliance with the ethical standards outlined in the 1964 Declaration of Helsinki and its later amendments. All participants provided written informed consent prior to their inclusion in the study. The study partnered with registered physicians providing prenatal care in metropolitan regions of Brno and Znojmo. Physicians conducted recruitment in their practices. Eligibility criteria included due date between March 1, 1991 and June 30, 1992 and residence in Brno or Znojmo regions. Self-report questionnaires were collected at 20 weeks of pregnancy (henceforth mid-pregnancy) from 4811 women, representing 60.9% of the total of 7895 births in Brno and Znojmo regions during the target period per the national registry (Piler et al. 2017). Follow-up questionnaires were collected in maternity hospitals within a week post-delivery and by mail at ten subsequent time points between child age 6 months and 19 years. Intervals between data collections ranged from 4 months to 4 years (*M* = 19.6 months). The present study uses data collected in mid-pregnancy (T1), after delivery (T2), at 6 months postpartum (T3), and at child age 7 years (T4), 11 years (T5), 15 years (T6), and 18 years (T7). Child psychological adjustment was assessed by mothers at T4, T5, T6, and T7. Women’s perinatal stress and depression were assessed at T1, T2, and T3. The main source of data missingness was attrition. Survey data were available from 3312 women at T4, 2609 women at T5, 1712 women at T6, and 1424 women at T7, resulting in a total 9057 possible person-period observations. Of these observations, 85% of women responded to questions concerning their child’s psychological adjustment across the waves. Additionally, 77% responded to perinatal surveys and had valid answers for stress instruments. Data on confounders were missing for roughly 10% of the remaining sample, leaving 5354 person-period observations.

### Measures

#### Main Predictors: Perinatal Maternal Stress Measures

Stress was assessed with three instruments administered at T1, T2, and T3. The first one, EPDS (Cox et al. 1987), is a valid measure assessing depressive symptoms (DS) during pregnancy and postpartum. The second instrument is a SLE count based on an inventory of 42 life events. The instrument was originally used in the ALSPAC birth-cohort study (Araya et al. 2009; Dewey et al. 1998; Dorrington et al. 2014; Dunn et al. 1998; Enoch et al. 2010; Hibbeln et al. 2007; Sullivan et al. 2013) and is described elsewhere (Stepanikova et al. 2018). Participants reported whether each event occurred during a specified time: conception to 20 weeks pregnant (T1), 20 weeks pregnant to delivery (T2), and delivery to 6 months (T3). The third instrument assesses the perceived stressfulness (PS) of life events. The instrument was based on the same 42-item list combined with the question, “How upsetting was this event?” ranging from not at all upsetting (0) to very upsetting (3). Events not experienced by respondents were coded as 0. All three measures of stress were treated as continuous.

#### Outcome: Child’s Emotional/Behavioral Difficulties (EBD)

Child’s EBD were assessed with the Strengths and Difficulties Questionnaire (SDQ), a validated instrument measuring hyperactivity, emotional symptoms, conduct problems, and peer problems (Goodman 1997, 2001). SDQ was mother-reported at ages 7, 11, 15, and 18 years.

#### Confounders

Maternal education and marital status were self-reported at T1. Age at delivery, child sex, low birthweight (< 2500 g), mode of delivery (cesarean section/other), and intrapartum complications (yes/no) were extracted from maternal medical records. Parity (nulliparous/other) and health behaviors (smoking [yes/no] at T1 and breastfeeding [yes/no] at T3) were self-reported. Women also reported their child’s SLE (count and PS) at ages 7–18 years.

### Statistical Analysis

For comparability, standardized measures of DS, SLE, PS, and SDQ were used to account for differences in wording and reporting period lengths across waves. To account for potential heteroskedasticity, the SLE, PS, and DS were transformed using a natural logarithm. Additional sensitivity analyses used terciles and quartiles to check for non-linearity. These analyses, available upon request, confirmed the results presented here. Given the transformations, the coefficients for perinatal stress should be interpreted as the following: a one percent change in the standard deviation of perinatal stress is associated with a change in the standard deviation of SDQ equivalent to the coefficient. While transforming the variables is necessary to make them comparable and to conform to model assumptions, the transformation leads to non-intuitive interpretation of the actual coefficients. As such, we focus our discussion to the relative size and direction of the coefficients. The dependent variable, SDQ, was modeled using growth curve models, a type of hierarchical linear modeling that accounts for correlations among the repeated observations nested within respondents. Time was measured as child’s age, centered at 7 years. Several model specifications were explored given past research and theoretical insight. Optimal model specification was determined using Bayesian Information Criterion (BIC) (Schwarz 1978) and Akaike Information Criterion (AIC) (Akaike 1974). BIC and AIC assess the overall model fit while taking into account the number of predictors. They allow comparison of non-nested models, such as ours. No significance test is associated with AIC and BIC but lower values indicate a better model fit. When comparing models, BIC difference of 0–2 provides weak evidence, 2–6 positive evidence, 6–10 strong evidence, and > 10 very strong evidence for a better fit of the model with the lower BIC value (Raftery 1995). Analyses were conducted using Stata 15 (StataCorp LLC: College Station, TX).

## Results

The mean mother-reported unstandardized SDQ scores were 9.02, 13.58, 7.42, and 6.74 for age 7, 11, 15, and 18, respectively. Both SLE and PS were highest during the first half of pregnancy (SLE, *M* = 3.36; PS: *M* = 5.48, Table 1). Conversely, DS was higher postnatally *(M* = 6.44) compared to the first (*M* = 6.33) and second (*M* = 5.88) half of pregnancy, suggesting that DS and SLE/PS tap into different constructs. All correlations among the examined stress measures were positive and statistically significant at p < 0.001, ranging from 0.18 to 0.87 in size (Table 2).

**Table 1:** Characteristics of the sample at child’s age 7 years (n = 1975), ELSPAC-CZ

	Mean	SE	%	SE
SDQ^a^	9.02	0.11		
*Maternal perinatal stress*^a^				
Maternal stressful life events, count				
Pregnancy, first half	3.36	0.09		
Pregnancy, second half	2.79	0.08		
Delivery to 6 months	2.88	0.06		
Perceived stressfulness of life events				
Pregnancy, first half	5.48	0.20		
Pregnancy, second half	4.49	0.17		
Delivery to 6 months	4.46	0.12		
Depressive symptoms				
Pregnancy, first half	6.33	0.10		
Pregnancy, second half	5.88	0.10		
Delivery to 6 months	6.44	0.10		
*Maternal background*				
Mother married (%)^a^			89.5	0.01
Maternal education (years)^a^	12.30	0.05		
Maternal age at delivery (years)^b^	25.96	0.11		
Nulliparous (%)^a^			51.3	0.01
*Delivery characteristics*^b^				
C-section (%)			8.0	0.01
Intrapartum complication (%)			23.3	0.01
Low birthweight (< 2500 g, %)			4.0	0.00
*Maternal health behaviors*^a^				
Breastfed at 6 months (%)			27.0	0.01
Smoked during pregnancy (%)			19.6	0.01
*Child characteristics*				
Gender: boy (%) ^b^			51.1	0.01
Child’s stressful life events count, age 7 years^a^	2.70	0.05		
Child’s perceived stressfulness of life events, age 7 years^a^	2.72	0.13		

*SDQ* Strengths and Difficulties Questionnaire, *SE* standard error

^a^Mother-reported

^b^Medical chart review

**Table 2:** Correlation coefficients among measures of perinatal maternal stress, ELSPAC-CZ

	Stressful life events, count^a^			Perceived stressfulness^a^			Depressive symptoms^a^		
	Pregnancy, first half	Pregnancy, second half	Delivery to 6 months	Pregnancy, first half	Pregnancy, second half	Delivery to 6 months	Pregnancy, first half	Pregnancy, second half	Delivery to 6 months
Stressful life events, count^a^									
Pregnancy, first half		0.26***	0.42***	0.87***	0.30***	0.39***	0.30***	0.20***	0.23***
Pregnancy, second half			0.35***	0.23***	0.76***	0.29***	0.18***	0.27***	0.21***
Delivery to 6 months				0.40***	0.40***	0.86***	0.22***	0.22***	0.28***
Perceived stressfulness^a^									
Pregnancy, first half					0.34***	0.42***	0.33***	0.22***	0.23***
Pregnancy, second half						0.43***	0.25***	0.37***	0.28***
Delivery to 6 months							0.26***	0.25***	0.30***
Depressive symptoms^a^									
Pregnancy, first half								0.46***	0.48***
Pregnancy, second half									0.55***
Delivery to 6 months									

***p < 0.001 (two-tailed tests)

^a^Standardized, logged. *N* = 1975

Table 3 shows results of growth curve models predicting SDQ scores from age 7 to 18 years. All measures of stress are standardized and logged; thus, coefficients are comparable within models. In Model 1, higher SDQ scores in childhood/adolescence are predicted by higher maternal SLE counts across all three perinatal periods (pregnancy, first half, b = 0.12, 95% CI [0.07, 0.17]; pregnancy, second half, b = 0.04, 95% CI [0.04, 0.06]; postpartum, b = 0.07, 95% CI [0.01, 0.13]). Higher maternal DS for the second half of pregnancy (b = 0.07, 95% CI [0.01, 0.13]) and postpartum (b = 0.09, 95% CI [0.03, 0.15]) is associated with higher SDQ. Model 2 replaces SLE count with maternal PS. PS scores in pregnancy are linked to higher SDQ (first half, b = 0.12, 95% CI [0.07, 0.17]; second half, b = 0.04, 95% CI [0.04, 0.06]). DS retains statistical significance for postpartum only (b = 0.10, 95% CI [0.04, 0.16]). In Model 3, postpartum DS (b = 0.09, 95% CI [0.03, 0.15]) and postpartum SLE count (b = 0.11, 95% CI [0.01, 0.21]) retain statistical significance along with PS for the second half of pregnancy (b = 0.09, 95% CI [0.01, 0.17]). Notably, the comparison of BIC statistics across all three models favors Model 1. BIC differences of 8.42 (Model 1 vs. Model 2) and 20.19 (Model 1 vs. Model 3) indicate strong and very strong evidence, respectively, for a better fit of Model 1. Based on these results, inclusion of SLE count along with DS yields the best model fit.

**Table 3:** Growth curve analysis for Strengths and Difficulties Questionnaire score (standardized) from age 7 to 18 years, ELSPAC-CZ

	Model 1		Model 2		Model 3	
	b	95% CI	b	95% CI	b	95% CI
Stressful life events, count^a^						
Pregnancy, first half	0.12***	[0.07, 0.17]			0.09	[0.00, 0.18]
Pregnancy, second half	0.04***	[0.02, 0.06]			0.03	[− 0.01, 0.05]
Delivery to 6 months	0.07*	[0.01, 0.13]			0.11*	[0.01, 0.21]
Perceived stressfulness^a^						
Pregnancy, first half			0.16***	[0.08, 0.23]	0.05	[− 0.09, 0.18]
Pregnancy, second half			0.13***	[0.07, 0.19]	0.09*	[0.01, 0.18]
Delivery to 6 months			0.04	[− 0.04, 0.12]	− 0.08	[− 0.22, 0.05]
Depressive symptoms^a^						
Pregnancy, first half	0.03	[− 0.02, 0.09]	0.03	[− 0.03, 0.08]	0.03	[− 0.02, 0.09]
Pregnancy, second half	0.07*	[0.01, 0.13]	0.06	[− 0.01, 0.12]	0.06	[0.00, 0.12]
Delivery to 6 months	0.09**	[0.03, 0.15]	0.10***	[0.04, 0.16]	0.09**	[0.03, 0.15]
AIC	13484.7		13493.1		13485.1	
BIC	13642.7		13651.2		13662.9	

Models adjust for maternal education, marital status, age at delivery, child gender, birthweight < 2500 g, mode of delivery, intrapartum complications, parity, smoking during pregnancy, breastfeeding at 6 months, and child SLE at ages 7–18 years

*b* unstandardized coefficient, *CI* confidence interval, *BIC* Bayesian Information Criterion, *AIC* Akaike Information Criterion

^*^p < 0.05, **p < 0.01, ***p < 0.001 (two-tailed tests)

^a^Standardized, logged

## Discussion

We presented the first prospective study comparing different survey measures of maternal stress taken at multiple times during pregnancy and postpartum. Each measure independently predicted long-term EBD of the child. Maternal postpartum DS yielded consistent associations across models, and SLE-based measures explained an additional part of variation. Results suggest that inclusion of SLE is valuable for capturing an aspect of stress not adequately assessed by DS alone. Used together, DS and SLE offer optimum prediction of offspring’s future psychological adjustment among the examined measures of stress. Other predictors of child maladjustment, such as harsh and inconsistent discipline, may be important additional mediators but were not measured here. Effect sizes for SLE-based measures decreased between early pregnancy and postpartum. In studies of maternal stress and child adjustment, SLE-based measures may be useful during pregnancy, while psychologically oriented measures such as DS may perform better postpartum.

Strengths of the study include its prospective longitudinal design, population-based sample, large sample size, and the use of validated survey instruments. The measurement of stress at three time points during pregnancy/postpartum is an important contribution. Few prior studies have compared life stress in pregnancy vs. postpartum, and, to our knowledge, none of them have differentiated between stress levels during different periods of pregnancy. In the present study, no single period stands out as *the* sensitive period, although some differences in effect magnitudes are evident. Supplemental analyses using interaction effects explored whether stress levels during earlier stages of pregnancy potentiated the association between stress during later pregnancy, after delivery, and child’s SDQ. Non-significant results suggest that they did not. At each observation period, stress influenced EBD of offspring regardless of how much stress the woman experienced at earlier and later observation periods.

These results must be interpreted in the light of study limitations. Sample attrition, a limitation of most cohort studies, is similar to other longitudinal surveys (Tourangeau et al. 2005). The baseline sample was demographically similar to the entire population of births in Brno and Znojmo regions (Piler et al. 2017) but generalizability to other populations is unknown. Results may not apply to pregnancies terminated before the fourth month, when the recruitment took place, and to still births, since all included women delivered a surviving child. The overarching goal of ELSPAC-CZ is investigating factors in maternal/child health. This goal is compatible with the present study; nevertheless, the data were not collected to answer the specific research questions considered here. Slight differences in the measurement of SLE across time points to achieve age-appropriateness (e.g., inclusion of additional age-relevant events) and simplify the survey (e.g., reducing the number of response categories) required standardization for comparability across instruments. SDQ, the main outcome, is a validated instrument relying on maternal report; therefore it is subject to bias inherent in maternal reports. For instance, maternal perceptions of child may vary by maternal depressive tendencies. As in any observational study, unmeasured confounding remains as a possibility despite statistical adjustment for a number of confounders.

The thrust of this investigation is methodological, but it also extends substantive evidence on long-term adverse outcomes of perinatal stress for child health. It considers the timing of stress exposure during the perinatal period and suggests that answers to the question whether stress is more harmful during pregnancy or postpartum depends on how stress is measured. In our best-fitting model, the magnitude of the effect of life events was the largest for early pregnancy. It was almost double compared to later pregnancy and triple compared to postpartum. Notably, the pattern was reversed for depressive symptoms. No effect was found for early pregnancy and the effect for postpartum was slightly larger compared to late pregnancy. Based on these results, life events matter most during early pregnancy while depressive symptoms are especially important during postpartum. We speculate that differential bio-psychological mechanisms link maternal stress during pregnancy vs. postpartum to child development. Stress hormones cross placenta and affect fetal neuro-development through inflammation (Osborne 2018) and epigenetic changes, such as DNA methylation of fetal genes involved in the stress response (Palma-Gudiel et al. 2013). In utero, exposure to stress may be important in and of itself, regardless of maternal affective response. During postpartum, the influence of stress on the child is heavily channeled through mother-infant interaction. Mother-infant bonding, high-quality caregiving, and breastfeeding are related to optimal emotional/cognitive development of the child (Victora et al. 2016), but these factors are compromised among depressed mothers (Tarullo et al. 2017; Beck et al. 2011).

The presented evidence can inform policies supporting women’s and children’s health. Past research advocates incorporating stress screening into pregnancy/postpartum care (Avalos et al. 2019). Our results reinforce this recommendation with evidence that each examined aspect of life stress plays an independent role in emotional adjustment during later childhood and adolescence. Exposure to SLE can be easily assessed in routine pregnancy/postpartum care to identify women at risk. To improve the effectiveness of screening, individual aspects of stress should be considered rather than conflating stress with depressive symptoms. Women with relatively high SLE exposure and depressive symptoms may benefit from further assessment, counseling, and treatment. On the level of society, population-based policies addressing poverty, unemployment, crime, victimization, and other contextual stress sources in women’s lives may benefit health of women and children.

In conclusion, we caution against uncritical replication of prior methodological choices in life stress research, particularly concerning DS-based measures. Empirically, postpartum EPDS performed well in relation to child EBD but for pregnancy, SLE-based measures outperformed DS. Theoretical and conceptual underpinnings of EPDS raise questions about its validity as an indicator of stress. DS could be linked to mother-reported child well-being through mechanisms that do not involve increased stress for the woman or fetus/infant. Physio-pathological processes related to a maternal depressive disorder may have specific implications for fetal development that differ from implications of maternal exposure to stressors. Additionally, antenatal and postnatal depression can relapse, increasing the risk of later-life exposure to maternal depression among children with maternal history of depression during pregnancy/postpartum.

Life stress during pregnancy/postpartum is a multi-faceted phenomenon not fully captured by any single survey instrument. Novel approaches for holistic stress assessment are needed in the future. Selection of currently available instruments should be informed by careful conceptual and methodological consideration. When a more comprehensive assessment of life stress is warranted, combining several types of measures may be helpful, although it must be weighed against increased respondent burden.
